# mSphereDirect: Author-Initiated Peer Review of Manuscripts

**DOI:** 10.1128/mSphere.00307-16

**Published:** 2016-12-12

**Authors:** Michael J. Imperiale, Thomas Shenk, Stefano Bertuzzi

**Affiliations:** a Department of Microbiology and Immunology, University of Michigan, Ann Arbor, Michigan, USA; b Department of Molecular Biology, Princeton University, Princeton, New Jersey, USA; c American Society for Microbiology (ASM), Washington, DC, USA

## EDITORIAL

A little over 1 year ago, the American Society for Microbiology (ASM) launched *mSphere* as an open-access, online, pan-microbial sciences journal. We established two major goals: publish cutting-edge science and implement policies and processes to make the publication experience less onerous for authors (1). During our first year, we believe that we have made excellent progress in both areas. We published 112 manuscripts. These articles have been downloaded extensively (>4,000 downloads/month), tweeted by authors and others, featured six times on “This Week in Virology/Microbiology/Parasitology,” and picked up by the media (*mSphere* has been mentioned more than 400 times in news outlets in the past year). The editors have strived to reduce the need for authors to engage in an ongoing back-and-forth dialog with reviewers and editors by aiming to limit revisions to one round. We have also sped up the turnaround on first decisions, on average letting authors know the disposition of their manuscripts within 3 weeks. All of these accomplishments are a testimony to a team of senior editors, editors, and staff members who believe strongly in our goals.

Through this process, we paid attention to the rapidly evolving publishing landscape and to the trends that affect the future of scholarly publishing. Most importantly, we identified key pressure points that, in general, dissatisfy the scientific community and asked ourselves if *mSphere* could be a journal of a kind like no other, one that addresses some of these issues in a creative way to improve the author experience while guaranteeing high-quality standards.

In our view, two major trends seem evident at the moment. First, there is a diffuse feeling that scholarly publishing is too slow and rigid and that the publication industry has not taken full advantage of the opportunities posed by the digital revolution, such as online collaboration and online communities. A key “biomarker” for this sentiment is the interest in preprints, which emerged recently in biology as an efficient way to communicate scientific results *before* peer review, by depositing manuscripts in a repository such as bioRχiv (2) (see http://www.asm.org/index.php/ceo-blog/item/8-preprint-publication-the-force-awakens-or-the-revenge-of-the-undead and asapbio.org). Preprints put the timing of communication of discoveries in the authors’ hands and not in those of editors and publishers. This is an interesting approach that ASM supports and that will significantly change the publication landscape. Today, peer-reviewed journal articles conflate two different purposes: communication of the discovery by publication of the article itself and establishment of relevance of the discovery, first through the peer review process and later through citations. With preprints, these two functions are split: discovery communication happens with posting of the preprint in advance of peer review, while relevance is assessed largely through peer review and citations of the published article, afterward (3).

The second element that we heard is that “peer review is broken,” with some even thinking that there is no value in peer review. While we deeply disagree that there is no value in peer review and in edited publications, we indeed feel that there is ample room for experimenting and improvement.

With these two elements in hand—preprints and needed innovation in peer review—we have decided to embark on an experiment to enhance the process, making it more agile and more transparent to authors by putting them in the driver’s seat. After much listening, discussion, and brainstorming, we are proud to announce a new pathway of manuscript review and submission for *mSphere*, called mSphereDirect. In a nutshell, mSphereDirect gives authors more control over the peer review process and, as such, introduces significantly more transparency and speed into the system.

Briefly, mSphereDirect will work as follows. Authors will write a manuscript, and they will ask two reputable scientists in the field, who have no conflicts, to act as reviewers. The authors will then consider the reviews, revise the manuscript in response to those reviews, and submit to the journal six items: the final manuscript, the two reviews, a cover letter that summarizes the review process the authors used, the authors’ rebuttal to the original reviewers, and an acknowledgment from the reviewers that they are satisfied with the final version. Then, an *mSphere* senior editor will make a rapid accept/reject decision about the manuscript. There will be no additional opportunities to revise the manuscript. In effect, then, authors will be responsible for ensuring that (i) they have chosen appropriate reviewers and (ii) they have carefully considered the content of the reviews. If accepted, the manuscript will be posted along with the names of the reviewers. Authors must ensure that the individuals they ask to review the manuscript agree to this requirement. As is the case now with any manuscript submitted to an ASM journal, authors can of course choose to post a preprint on a server at any time. Additionally, not only can authors submit their final manuscript to bioRχiv but the paper will be flagged as a pending publication by *mSphere* immediately, if accepted.

What are the advantages of mSphereDirect? First, it takes the mystery out of the peer review process, because authors, with clear policy guidelines, will be able to choose the people they judge to be most appropriate to serve as reviewers. This approach lessens the potential for an uninformed or arbitrary review, because the author will identify referees who know the topic and can appreciate the significance of the work. Second, peer review will become a more constructive experience, because the reviewers can provide candid advice to the authors. We strongly believe that the peer review process, when functioning properly, improves the quality of the science being presented. Third, it will significantly decrease the time between uploading of the manuscript to the journal and posting on the website: we will make a decision within five working days, and the paper will be posted within 4 weeks, which is our standard turnaround after a paper has been accepted. Fourth, one can think about mSphereDirect as the democratization of the American Academy of Microbiology submission track at *mBio*. Until now, this track has been the most rapid path to publication in an ASM journal; and, importantly, evidence shows that the quality of the papers published through this track has been indistinguishable from papers submitted through the standard track in which an editor oversees the review process (4).

Of course, there are potential issues that arise with this system. Let us state from the outset that we fully expect that authors and reviewers will adhere to the highest ethical standards while engaging in the presubmission process. Without the integrity of all involved, this system is not viable. We will therefore be providing clear guidance to authors and reviewers on what will be expected by the journal. ASM has also developed an ethics resources website to help authors understand the issues, enhance rigor, and facilitate publication in ASM journals (http://journals.asm.org/site/misc/ethicsportal.xhtml). Having the reviewers’ names posted with the article will also enhance the quality of the reviews. We hope that authors and reviewers will engage in an iterative process, as needed, to improve the quality of the science and the manuscript. We wish to state clearly, however, that if a senior editor is at all uncomfortable with either the choice of reviewers or the quality of the reviews, the manuscript will be rejected.

mSphereDirect will launch on January 11, 2017. Over the next year, we plan to tweak and improve the pathway as we gain experience with this new way of managing peer review.

Please read the FAQs posted on the journal website (http://msphere.asm.org/content/mspheredirect-faqs) before beginning the review process and submitting your manuscript. We are very interested in your thoughts as you engage in this experiment with the way ASM journals undertake peer review. Please contact Mike Imperiale if you have any question.

The regular review track of *mSphere* remains a very valuable option for those who wish to pursue the “classic” approach, but we also look forward to your participation in mSphereDirect, where you, as an author, are front and center. As we say at *mSphere*, it is all about you: your research, your direction, you’re in control.
